# Leaching losses from Kenyan maize cropland receiving different rates of nitrogen fertilizer

**DOI:** 10.1007/s10705-017-9852-z

**Published:** 2017-05-16

**Authors:** T. A. Russo, K. Tully, C. Palm, C. Neill

**Affiliations:** 1 Department of Geosciences and the Earth and Environmental Systems Institute, Pennsylvania State University, 310 Deike Building, University Park, PA 16802, USA; 2 Columbia Water Center, Columbia University, 500 West 120th St., New York, NY 10027, USA; 3 Department of Plant Science and Landscape Architecture, University of Maryland, College Park, MD 20742, USA; 4 Agriculture and Food Security Center, Lamont-Doherty Earth Observatory, Columbia University, Palisades, NY 10964, USA; 5 Department of Agricultural and Biological Engineering, University of Florida, Gainesville, FL 32611, USA; 6 Ecosystems Center, Marine Biological Laboratory, Woods Hole, MA 02543, USA; 7 Department of Ecology and Evolutionary Biology, Brown University, Providence, RI 02912, USA; 8 Woods Hole Research Center, 149 Woods Hole Road, Falmouth, MA 02540, USA

**Keywords:** Leaching, Nitrogen fertilizer, Nitrate, Numerical modeling, Sub-Saharan Africa

## Abstract

Meeting food security requirements in sub-Saharan Africa (SSA) will require increasing fertilizer use to improve crop yields, however excess fertilization can cause environmental and public health problems in surface and groundwater. Determining the threshold of reasonable fertilizer application in SSA requires an understanding of flow dynamics and nutrient transport in under-studied, tropical soils experiencing seasonal rainfall. We estimated leaching flux in Yala, Kenya on a maize field that received from 0 to 200 kg ha^–1^ of nitrogen (N) fertilizer. Soil pore water concentration measurements during two growing seasons were coupled with results from a numerical fluid flow model to calculate the daily flux of nitrate-nitrogen (NO_3_^–^-N). Modeled NO_3_^–^-N losses to below 200 cm for 1 year ranged from 40 kg N ha^–1^ year^–1^ in the 75 kg N ha^–1^ year^–1^ treatment to 81 kg N ha^–1^ year^–1^ in the 200 kg N ha^–1^ treatment. The highest soil pore water NO_3_^–^-N concentrations and NO_3_^–^-N leaching fluxes occurred on the highest N application plots, however there was a poor correlation between N application rate and NO_3_^–^-N leaching for the remaining N application rates. The drought in the second study year resulted in higher pore water NO_3_^–^-N concentrations, while NO_3_^–^-N leaching was disproportionately smaller than the decrease in precipitation. The lack of a strong correlation between NO_3_^–^-N leaching and N application rate, and a large decrease in flux between 120 and 200 cm suggest processes that influence NO_3_^–^-N retention in soils below 200 cm will ultimately control NO_3_^–^-N leaching at the watershed scale.-the daily flux of nitrate-nitrogen (NO_3_^–^-N). The lack of a strong correlation between NO_3_^–^-N leaching and N application rate, and a large decrease in flux between 120 and 200 cm suggest processes that influence NO_3_^–^-N retention in soils below 200 cm will ultimately control NO_3_^–^-N leaching at the watershed scale.

## Introduction

Hunger and malnutrition persist in many developing countries despite technological advances in agricultural food production and distribution during the last 50 years. Agricultural productivity in sub-Saharan African (SSA) has lagged behind the rest of the world (Hazell and Wood 2008; Monfreda et al. 2008) and provides motivation for the African Green Revolution (AGR), a movement that aims to increase food production by combining science, technology, and policy (Annan 2004). A key component of the AGR is to increase the application of fertilizers from around 8 to 50 kg N ha^–1^ year^–1^ (Denning et al. 2009; Sanchez et al. 2009). Future increases in fertilizer application in SSA are expected because fertilizer use is currently many times lower than recommended rates in most smallholder farms, and even the recommended rates are far less than rates of fertilizer application in most developed countries (Vitousek et al. 2009).

While there is substantial evidence that fertilizer application will increase crop yield (Sanchez et al. 2007; Denning et al. 2009; Nziguheba et al. 2010; Snapp et al. 2010; Sanchez 2015; Mafongoya and Jiri 2016), excess fertilizer application does not improve yield and can have environmental and public health consequences (Goulding 2000; Ju et al. 2009). Excess N in agroecosystems can increase concentrations of nitrate-nitrogen (NO_3_^–^-N) in ground and surface waters and cause algae blooms, fish kills, and risks to public health (Carpenter et al. 1998; Howarth et al. 2002; Rabalais et al. 2002; Galloway et al. 2003). The presence of NO_3_^–^-N in drinking water is particularly harmful to infants, pregnant women, and certain populations with hereditary blood diseases (Knobeloch et al. 2000; Gatseva and Argirova 2008). For developing regions where drinking water is often obtained from shallow wells or streams, these risks are particularly acute. In addition to environmental and public health costs, over-application of N fertilizer represents an economic burden on smallholder farmers.

Plant nutrient uptake and N losses from farm fields are a function of several environmental and management variables, including soil texture, soil mineralogy, crop type, tillage method, climate and/or irrigation scheduling, and N fertilizer type, application timing, rate, and placement. Enhancing plant N fertilizer uptake efficiency through agronomic practices can significantly reduce N losses from the system. Soil type controls nutrient soil solution flux by the degree to which it retains ions and inhibits fluid flow, particularly in response to seasonal and pulsed rainfall. Clay-rich soils tend to have lower leachate loss rates than coarser textured soils and because of greater residence time of nutrients in soil solution and lower total flux (Simmelsgaard 1998), but aggregation of clays, particularly weathered clays of tropical soils, can also lead to very high infiltration rates (Palm et al. 2007; Scheffler et al. 2011). Clay-rich soils also will typically retain more base cations than sandy soils of the same mineralogy because clays contribute to fixedcharge cation exchange capacity (Brady and Weil 2007). However, in weathered tropical soils, the presence of variable charge clays like kaolinite, hematite, and gibbsite can promote anion exchange capacity (AEC) and thus the adsorption of NO_3_^–^ (Singh and Kanehiro 1969; Kinjo and Pratt 1971). Because these weathered tropical soils can reach depths of many meters or even tens of meters, processes of nutrient attenuation and cycling can occur well below crop rooting depths. The paucity of field leachate measurements on sub-Saharan soils make it difficult to predict how much N will be lost from farm fields as N fertilizer applications increase across Africa.

Estimation of NO_3_^–^-N leaching requires measurement of soil solution NO_3_^–^-N concentrations and the estimation of soil water movement. Many studies have examined the advantages and disadvantages of different field methods for measuring soil solution in situ, but there is no standard method (Zhu et al. 2003; Siemens and Kaupenjohann 2004; Fares et al. 2009; Meissner et al. 2010). For example, tension lysimeters provide access to deep soil horizons, and can be sampled daily for near real-time data on soil solution chemistry (Tully and Weil 2014). In contrast, subsurface drains or pans are a more direct methods of measuring NO_3_^–^-N flux (Lamba et al. 2013), but they are highly invasive and can require significant earthwork. Soil extractions, such as with potassium chloride, cannot easily measure concentrations from the same point over time and may measure NO_3_^–^-N that is tightly held and not moving in solution. These sample collection methods may bias subsequent flux estimates by preferentially sampling from separate subsurface pools of NO_3_^–^-N. For example, comparisons of NO_3_-N concentrations from lysimeters and those derived from soil extractions (representing all soil pores) suggest that lysimeters can underestimate concentrations because they cannot sample from higher NO_3_^–^-N concentrations in disconnected pore spaces (Djurhuus and Jacobsen 1995; Darrouzet-Nardi and Weintraub 2014). Alternatively, if there are dominant macropores in the system, lysimeters may over-represent bulk soil pore water concentrations rather than capturing potentially dilute leachate traveling through rapid drainage pathways (van der Laan et al. 2010).

Point measurements of soil solution NO_3_^–^-N concentration can be used with soil water balance models to estimate soil solution flux, or the entire NO_3_^–^-N solute transport system can be modeled (Ajdary et al. 2007; Perego et al. 2012; van der Laan et al. 2014). Mechanistic models solve physics-based transport equations for fluid flow through variably saturated soils, typically based on the Richards Equation, as in VS2D (Lappala et al. 1987) and HYDRUS (Tafteh and Sepaskhah 2012), or the Green-Ampt Equation. Mechanistic models can be computationally intensive, however they capture the time dependence and spatial variability of water fluxes. Comparisons of NO_3_^–^-N flux models illustrate strengths of specific models for certain cropping and soil conditions (Moreels et al. 2003; Groenendijk et al. 2014). Noted weaknesses include typical omission of certain transport phenomena such as multi-phase flow, hysteresis, and difficulty predicting nutrient transport in low N fertilizer application settings. Advances in predictive nutrient flux modeling account for N transformations and uptake, organic matter mineralization, and crop yield (Endo et al. 2009; Nolan et al. 2010), though model input requirements are greater than for fluid flow models.

Very little data exist on the potential impacts of increased fertilizer applications on shallow groundwater quality across the diverse agroecological zones and soil types that span the African continent. To date, only five studies measured leachate concentrations in pore water from maize fields in SSA (Poss and Saragoni 1992; Kamukondiwa and Bergstrom 1994; Nyamangara et al. 2003; Mapanda et al. 2012). In fact, more data exist on NO_3_^–^-N leaching from individual research farms in Central California (e.g., West Side Field Station), than from all of SSA combined.

Previous studies from sub-Saharan sites demonstrate leaching variability across soil types and over time, typically for sites with short histories of adequate fertilizer application. Two studies on the same research farm with loamy fine sands in Togo reported large differences in fertilizer loss rates. The first study found higher N leaching losses (*35% at 150 cm), and lower maize N uptake (Poss and Saragoni 1992) compared to a later study where leaching losses were low (*5% at 100 cm), while maize N uptake was nearly double the N application rate (Sogbedji et al. 2006). This disparity may result from differences in measurement methods, tension lysimeter versus monolith lysimeters, respectively, or other crop management practices. Conversely, three studies in Zimbabwe found similar fertilizer leaching loss rates perhaps due to the fact that a similar method was employed (repacked, gravity draining lysimeters at 100 cm). All three studies were conducted near Harare, and found fertilizer N losses around 15% from sandy loams (Kamukondiwa and Bergstrom 1994; Nyamangara et al. 2003; Mapanda et al. 2012) and 12% from clays (Mapanda et al. 2012). In Kenya, a study on a clayey soil measured leaching losses by differences in soil N between depth layers, and estimated that roughly 19% of added N was lost below 80 cm in maize systems (Kimetu et al. 2006).While NO_3_^–^-N leaching tends to increase with N fertilizer application rates in temperate agricultural systems (Sogbedji et al. 2000; Perego et al. 2012), clay-rich, tropical soils may have relatively low leaching losses because of high anion exchange capacity and low permeability. Sogbedji et al. (2006) found a correlation between N application rate and NO_3_^–^-N leaching losses on loamy fine sands in Togo, however only during one season using two treatments. The coupled system of hydrology-agronomy-geochemistry is under-studied in tropical soils in SSA, making it difficult to predict and manage nutrient flow and transport dynamics where increased N fertilizer use will occur.

This study examined N loss rates from N fertilized maize in clay-rich, deep tropical soils in western Kenya. We used a simple and robust form of mechanistic modeling to improve our understanding of NO_3_^–^-N dynamics in response to rainfall and N fertilizer application. The objectives were to: (1) measure pore water NO_3_^–^-N concentrations and soil moisture content on plots with a range of N fertilizer application rates, (2) calculate NO_3_^–^-N flux below the maize root zone using numerical modeling, and (3) evaluate NO_3_^–^-N fluxes with respect to nutrient application rates and rainfall patterns over a 2 year study period. These results will contribute to a small but critical body of literature on nutrient and soil water transport, and response to increased N fertilizer use in SSA.

## Materials and methods

### Field location

**Fig. 1:**
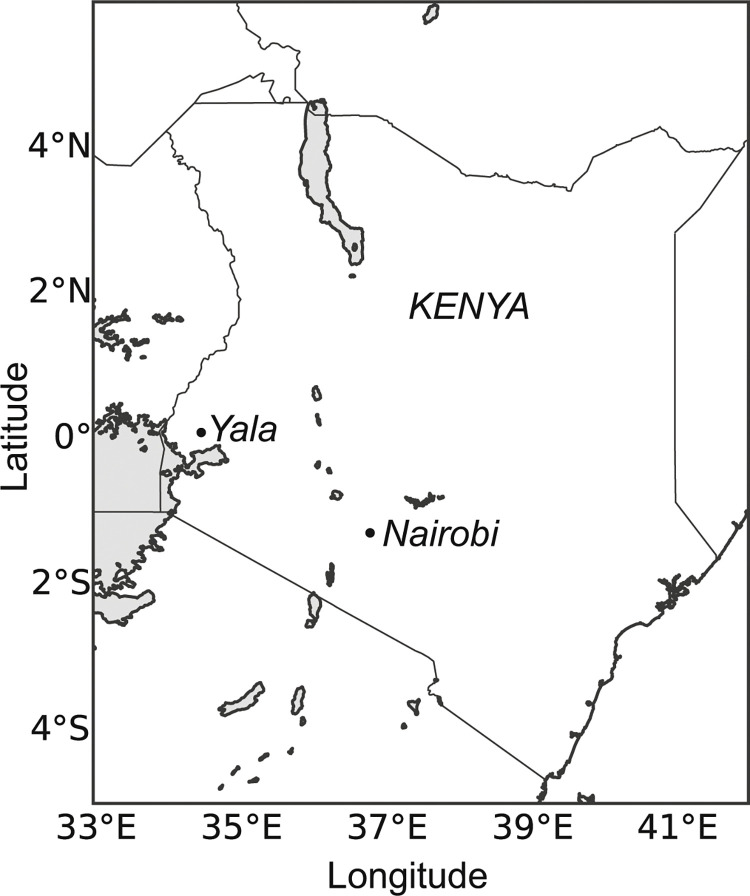
Location of Yala study area in Kenya

This study was conducted in Yala (0°5′42.99″N, 34°32′5.63″E) in the western Kenyan highlands(Fig. 1). The region receives 1816 mm of precipitation per year in two rainy seasons on average; the “long rains” extend from March through June and the “short rains” extend from October to November or early December. Precipitation for the two growing seasons (April to August) was 1162 mm in 2013 and 760 mm in 2013. The mean annual temperature is 23.5 °C (Nziguheba et al. 2010; Palm et al. 2010). The region is characterized by rain-fed maize-based agricultural systems (Dixon et al. 2001).

**Fig. 2:**
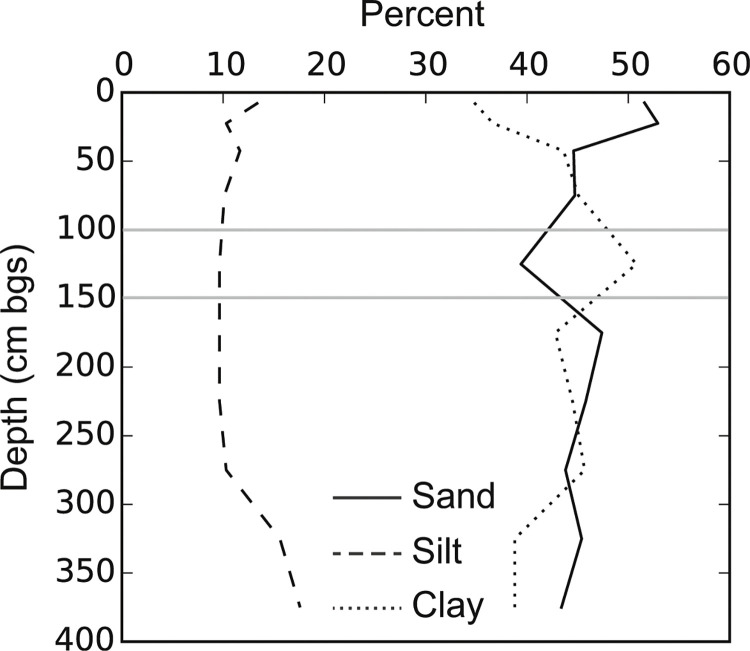
Average percent sand, silt, and clay below ground surface (bgs) for the upper 4 m of soil. The *horizontal gray lines* represent the boundaries of the three modeled soil layers; the model domain extends to 500 cm bgs

The field area has well-drained sandy clay loams of oxidic mineralogy (Eutric Ferralsol). Soils are about 36% clay in the top 30 cm of soil with slightly higher average clay content (44%) in the subsoils (Fig. 2). These soils are derived from fertile volcanic parent materials but are low in C, N, and P after decades of low input cultivation, similar to many soils across SSA (Smaling et al. 1996; Palm et al. 1997). Topsoils (0–15 cm) have 15.4 cmol 100 g^–1^ effective cation exchange capacity (ECEC), 1.9% organic C, and 0.11% total N (Table 1) (Tully et al. 2016; Almaraz et al. in prep). We conducted our field experiments on lands owned by the Kenya Broadcasting Company Nyamninia in Yala, Kenya. The site was converted to agriculture in the 1960s or 1970s. Fields were left fallow from 1979 to 1989 and from 1994 to 2007; in other years, maize, beans (multiple genera within the *Fabaceae*), and sweet potatoes (*Ipomoea batatas* (L.) Lam.) were grown by local farmers without mineral nitrogen applications.

**Table 1:** Soil properties (0–15 cm) in Yala, Kenya (Tully et al. 2016)

Measurement	Value
pH_water_	5.97 (0.13)
P (μg/g)	0.06 (0.005)
K (mg/g)	50.5 (0.21)
Ca (mg/g)	19.40 (1.06)
Mg (mg/g)	2.26 (0.11)
EC salts (μS/cm)	248.75 (22.89)
ECEC (meq/100 g)	15.4 (0.45)
Org C (%)	1.90 (0.08)
Total N (%)	0.11 (0.00)

Values in parentheses are the standard error of the mean

### Nitrogen application rate experimental design

We used a randomized complete block design (RCBD) to determine the effects of increasing N fertilizer on soil solution flux. The experiment had six application rates of inorganic N fertilizer: 0, 50, 75, 100, 150, and 200 kg-N ha^–1^. However, this study only focuses on the 0, 50, 75, and 200 kg-N ha^–1^ application rates. Each treatment had four replicates for a total of 16 plots. Mineral N fertilizer was applied basally in a split application: one-third at planting (using diammonium phosphate; (NH_4_)_2_HPO_4_) and the remaining two-thirds 5 weeks later (as urea; CO(NH_2_)_2_). Smallholder maize is typically fertilized at low levels (< 10 kg N ha^–1^), but the Alliance for a Green Revolution in Africa (AGRA) currently promotes fertilizer application rates of 50–75 kg N ha^–1^. In this study we aimed to capture the range of potential fertilizer rates (50–75 kg N ha^–1^) as well as a high rate (200 kg N ha^–1^) characteristic of intensified maize farming in the Midwestern US.

Maize (*Zea mays* L.) was planted at 30 × 75 cm spacing (Kenya Seed Company WH403). Plots were 3 m × 6 m, with a total of 80 plants per plot, with the outer two plants on all sides serving as “buffer plants” between plots. We assumed no lateral flow between plots due to the flat topography, the buffer rows of maize, and no overland flow was observed. See Hickman et al. (2015) for full plot design.

### Climate and soil data collection

A weather station (Campbell Scientific) was placed at the field site in November 2011. Relative humidity and air temperature sensors were installed at 2 m above the ground, and wind speed and solar radiation at 4 m. Rainfall was measured using a tipping bucket gauge. Meteorological values were recorded every 30 s and averaged every hour with a data logger (CR800, Campbell Scientific). Solar radiation was measured using a net radiometer (NR-Lite2, Kipp and Zonen, Inc. Delft, The Netherlands), which measured both short-and long-wave radiation.

We measured bulk density with a slide hammer using a stacked-ring method (core volume = 205.9 cm^3^; Core Sampler Complete, AMS Idaho, USA). In May of 2012, soil profiles were collected to 400 cm and divided into 10 depth segments: 0–15, 15–30, 30–50, 50–100, 100–150, 150–200, 200–250, 300–350, and 350–400 cm in each unfertilized plot. Composite samples from all depth segments were analyzed for soil texture using the hydrometer method (Bouyoucos 1962).

Soil moisture content was measured using time domain transmissometry (TDT) sensors (Acclima, Inc.) at 120, and 200 cm below ground surface (bgs). Measurements were made from August 5 to November 13, 2013 in three plots receiving either 0 or 75 kg N ha^–1^ year^–1^, representing the current and AGRA recommended N fertilizer application practices, respectively. The sensors measured volumetric soil moisture content every 20 min, averaged over a 100 mL volume. Measurements from all three plots showed the same magnitude and timing of soil moisture changes associated with precipitation events, suggesting that one fluid flux model could be applied for all N fertilizer application rates. Masaka et al. (2013) also found no significant difference between leachate volumes across N fertilizer treatments when applied to the same soils, though NO_3_^–^-N soil solution flux varied due to differences in concentration. Soil moisture content time series data from the sensors were used to calibrate a fluid flow model.

### Nitrate sample collection and selection

Three ceramic cup lysimeters (SoilMoisture Equipment Corp., Goleta, CA 0653 × 01-B0.5M2; inner diameter of 4.2 cm; 0.5 bar; Alumina body) connected to a 1–1.5″ schedule 40 PVC pipe using epoxy. Lysimeters were installed at 15, 120, and 200 cm in plots receiving 0, 50, 75, and 200 kg N ha^–1^ year^–1^ within 15 cm of a maize plant (Tully and Weil 2014) in January of 2012. This method assumes that samples obtained from the lysimeters are representative of the average leachate concentration. The 120 and 200 cm depths correspond to soil moisture measurements and are intended to represent within and beneath the maize root zone, respectively. We augered holes (5 cm diameter) to each depth, installed lysimeters, then backfilled soil around the holes to prevent movement of water along the PVC pipes. Pipes were fitted with a rubber one-hole plug through which a siphon tube was inserted. This study used only the 120 and 200 cm depth data, which coincided with depths of the soil moisture measurements. The day before sampling, lysimeters were purged of any water and an internal pressure of –0.05 to –0.06 MPa was applied. Soil solution samples were collected prior to maize planting in 2012 and 2013, daily for 3–5 days following planting (5 April 2012 and 10 April 2013), and weekly until the second N fertilizer application (7 May 2012 and 8 May 2013). Soil solution samples were collected 3–5 days following the second N fertilizer event, then weekly for 4 weeks, and bi-weekly until harvest (28 August 2012 and 16 August 2013) for a total of 25 collection periods across the growing season giving us high temporal resolution soil solution data. In total, sampling was conducted from April 2012 to December 2013, though not every lysimeter produced water at each sampling time.

### Chemical analysis

Soil solution samples were kept in acid-washed (5% HCl) high-density polyethylene bottles to which a pinch of Thymol (5-methyl-2-[1-methylethl]phenol; Acros Organics) was added to inhibit biological activity. Within 3 days of collection, unfiltered water samples were analyzed for NO_3_^–^-N using an ionselective electrode (ISE; Horiba, Inc. B-342; Kyoto, Japan) in Kenya. The ISE has a two-point calibration (6.8 and 68 mg L^–1^), and was calibrated every 10 samples, and each sample was analyzed in triplicate with the mean reported. The ISE method has high agreement (*r*^2^ = 0.96) with standard colorimetric method for NO_3_^–^-N analysis (Tully and Weil 2014).

Soil solution samples were also transported to the Marine Biological Laboratory (Woods Hole, MA) and solution samples that exceeded 70 mg NO_3_^–^-N L^–1^ (upper range of the ISE) were analyzed on a LACHAT QuikChem (LACHAT Instruments Loveland, CO) using cadmium-reduction. Extracts were diluted as necessary if they exceeded the highest calibration standard that was within the detectible range of the colorimeter.

Because of variability between sample NO_3_—N concentrations taken from replicate plots with the same N fertilizer treatment, we applied the Kolmogorov–Smirnov test to compare observations and identify anomalous sets of observations. The sample set from each plot of a given N fertilizer treatment was compared to the other three replicate plots using the Kolmogorov–Smirnov test, a nonparametric test used to test the null hypothesis that two sample sets come from the same distribution. We rejected the null hypothesis for *p* values <0.01. The entire plot record was omitted from the analysis if the data was significantly different than two or more of the other datasets from the same N fertilizer application level. One plot from each N fertilizer treatment group was identified as having a distinct distribution of observations compared to the other replicates and these plots were excluded from further analysis.

### Model development

We used the open source numerical model VS2D (Lappala et al. 1987) to calculate infiltration fluid flow through variably saturated soils (e.g., Russo et al. 2012). The model uses a finite difference method to approximate fluid flow based on the Richards Equation. VS2D was selected rather than a model that accounts for fate and transport, or one that includes crop uptake because our objective was to estimate leachate flux within and below the root zone during the study period. Solute transport and biophysical crop models simulate the full plant-nutrient-soil–water system, however additional model complexity is accompanied with greater uncertainty, especially where field measurements are limited. Using the measured pore water concentrations and modeled fluid flux at the same depth is a robust method for estimating NO_3_^–^-N soil solution flux, though it does not account for impacts of changing management or climate on crop nutrient uptake.

A 5 m depth model domain was used to simulate fluid flow in the 1-D vertical direction with a daily time-step. We used 115 stacked grid cells with 3 cm spacing from 0 to 3 m, 10 cm spacing from 3 to 4 m, and 20 cm spacing from 4 to 5 m. VS2D can have internal sinks and sources, and simulates evaporation and plant transpiration. Soil texture measurements (Fig. 2) were used to develop a model domain with three horizontal layers with distinct properties from 0 to 100, 100–150, and 150–500 cm. Because there was not significant variability between soil properties or soil moisture across the 16 maize plots, we used a single fluid flow model to represent fluid transport in all 16 maize plots. Surface boundary conditions were determined using measured daily total precipitation and calculated evapotranspiration (ET). The vertical boundaries on the sides of the model were defined as no-flow boundaries, and the horizontal boundary at the base of the model, 500 cm, was a gravity drain boundary, allowing gravity driven vertical flow out of the model domain. There was no evidence of the influence of a rising groundwater table. The VS2D model allows users to define the water retention curve using Van Genuchten or Brooks-Corey parameters, or their own measurements.

We used the surface boundary conditions (measured precipitation minus ET) to inversely model the Brooks-Corey unsaturated fluid flow parameters. The model parameters were calibrated using 7.5 weeks of observed soil moisture time series data at 120 and 200 cm spanning before and during the 2013 short rains. Porosity, saturated hydraulic conductivity, residual moisture content, and the Brooks-Corey soil moisture characteristic parameters were initially estimated based on soil texture, and were refined during model calibration. Daily simulation outputs for the study period for observation points (120 and 200 cm) included soil moisture content and vertical fluid velocity.

### Soil solution concentration data analysis and flux calculation

Modeled soil moisture content and modeled soil water transport velocities were combined with measured NO_3_^–^-N nutrient concentration measurements to calculate NO_3_^–^-N soil solution flux during the study period. Daily downward fluid flux, *q_t_* (m day^–1^), was calculated as the simulated daily downward fluid velocity *v_t_* (m day^–1^) multiplied by the daily soil moisture content, *h_t_* (Eq. 1). Daily vertical NO_3_^–^-N flux, *F_t_* (kg NO_3_^–^-N ha^–1^ day^–1^) is calculated as the daily fluid flux multiplied by the measured, or interpolated, NO_3_^–^-N concentration (mg L^–1^) (Eqs. 2, 3).

(1) $$q_{t}=v_{t}\theta_{t}$$

(2) $$F_{t}=10q_{t}\left(C_{i}\right),\text{ for }m_{i}=t$$

(3) $$\begin{array}{l} F_{t}=10q_{t}\left(C_{i}+(\frac{C_{i}-C_{i+1}}{m_{i+1}-m_{i}})\left(m_{i}-t\right)\right).\\ \text{for }t>m_{i} \end{array}$$

where *F_t_* is NO_3_^–^-N flux on day *t, C_i_* is NO_3_^–^-N concentration for measurement *i*, and *m_i_* is day (*t*) of each concentration measurement. NO_3_^–^-N flux was calculated by multiplying fluid flux by concentration for days when concentration was measured (Eq. 2). For all other days, the concentration was assumed to vary linearly between measurements over time (Eq. 3). Positive *F_t_* values indicated downward soil solution flux, and the coefficient of 10 produces flux in units kg N ha^–1^. The fluxes reported were calculated from the mean NO_3_^–^-N concentration value from the replicate plots of each N fertilizer application rate.

## Results and discussion

### Pore water concentrations

**Fig. 3:**
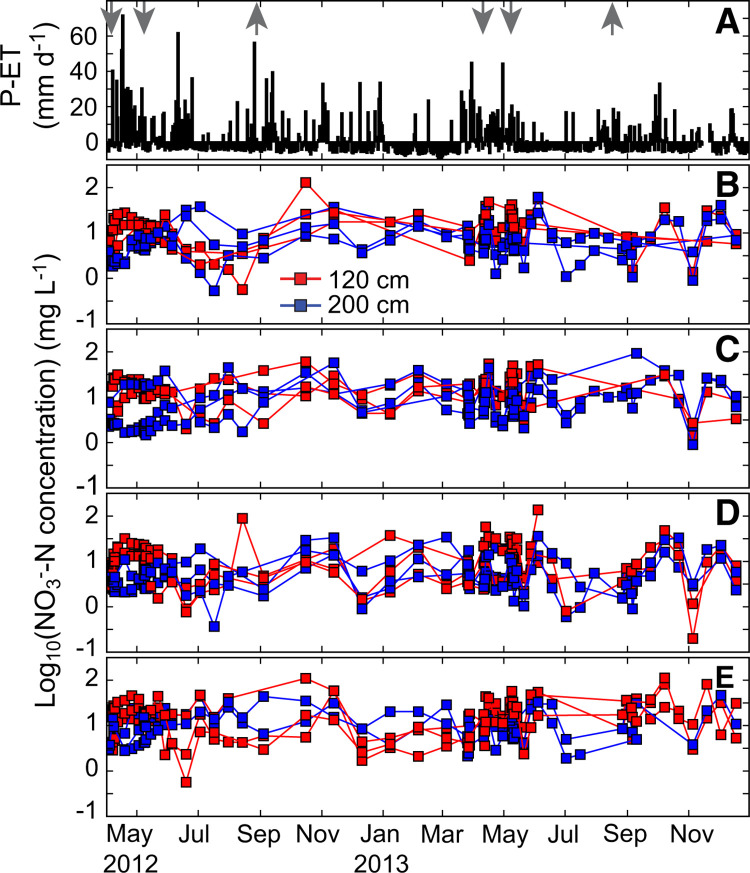
**a** Daily net precipitation minus ET, *down-arrows* indicate fertilizer application and *up-arrows* indicate harvest, **b** measurements of NO_3_^–^-N concentration measurements at 120 (*red*) and 200 cm (*blue*) for fertilizer applications rates of **b** 0, **c** 50, **d** 75, and **e** 200 kg-N ha^–1^. *Lines* connect data from individual replicate plots. (Color figure online)

Soil pore water NO_3_^–^-N concentrations ranged between 0.37 and 130 mg L^–1^, with the highest concentration measured on a plot receiving 0 kg N ha^–1^. There was a weak correlation between pore water NO_3_^–^-N concentrations and N fertilizer application rate at 120 cm (R^2^ = 0.28), and no correlation below the root zone at 200 cm (R^2^ = 0.04) (Fig. 3). The measurements for each set of N fertilizer application rate and depth were right skewed with a few high concentrations, typically corresponding to periods of heavy rainfall. The difference in concentration with depth was most notable following fertilization at the beginning of the wet season (Fig. 3). Pore water NO_3_^–^-N concentrations rose quickly at 120 cm following the start of the rains, while concentrations measured at 200 cm tended to rise slowly throughout the growing season. Following harvest, NO_3_^–^-N concentrations at both depths generally rose through September and October during the beginning of the short rains, likely due to downward transport of remaining applied N fertilizer in the soil, and mineralization of organic matter in the soil followed by nitrification of the applied ammonium in the wet and warm conditions. Following the short rains, NO_3_^–^-N concentrations declined through the dry season before the long rains began in March.

Using daily concentration values from Eq. 3 for the growing season, we find that average pore water NO_3_^–^-N concentrations were highest on plots receiving 200 kg N ha^–1^ at 120 cm for both growing seasons and at 200 cm in the 2012 growing season (Table 3). The pore water NO_3_^–^-N concentrations were approximately 1.4 times higher in samples from 120 cm (17 mg L^–1^) compared to 200 cm (12 mg L^–1^). Though generally highest on the high N fertilizer treatment plots, average NO_3_^–^-N concentrations did not correlate with N application for all treatments. Pore water NO_3_^–^-N concentrations were higher for all plots during 2013 compared to 2012 at 120 cm, while only half of the plots had higher concentrations during 2013 at 200 cm (Table 3). Higher pore water concentrations in 2013 may be due to lower crop nutrient uptake within the root zone because of drought (Tully et al. in preparation) and decreased flushing of mineral N due to less precipitation.

During both growing seasons, the NO_3_^–^-N concentrations varied within the four replicate plots that received the same N fertilizer application treatment rate (Fig. 3). The largest range of measurements across plots with the same N fertilizer treatment taken on a single day was 109 and occurred in a plot receiving 200 kg N ha^–1^. We reduced the variation before estimating NO_3_^–^-N flux by omitting data from statistically distinct plots determined with the Kolmogorov–Smirnov test across replicate plots. Anomalous measurements from one of the replicate plots may have been caused by small-scale heterogeneities or differences in lysimeter intersection with macropores. Lysimeters sample fluids in the macropores, the matrix, or a combination of the two. If the lysimeters did not intersect any macropores, they may misrepresent nutrient concentrations in the well-connected pore spaces (Booltink 1995; Fares et al. 2009). The difference in concentrations between macropore and matrix water may be attributed to varying connection to high or dilute surface water concentrations, and different residence times in macropores affecting the opportunity for denitrification (Schmidt et al. 2011). Adding wetting front detectors (van der Laan et al. 2010), increasing the number of lysimeter replicates within the same plot, or installing a pan or monolith lysimeters to capture the total drainage water would potentially help estimate a more precise average. However, there are land disturbance, labor, cost, and space constraints associated with these alternatives, and monolith lysimeters also require long rest periods (~ 16 months) before they provide accurate measures of soil solution quantity or chemistry. Despite these barriers, future studies in SSA, especially on fields designated for research, may consider employing these methods.

### Leachate fluxes

#### Hydrologic model development

**Fig.4:**
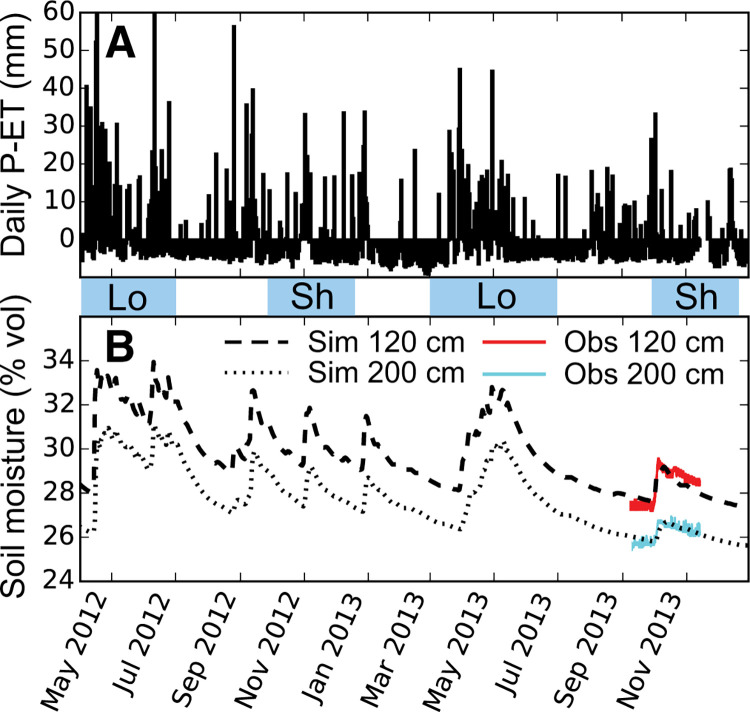
Model calibration results. **a** Daily net precipitation minus ET, **b** soil moisture content simulated (*dashed* and *dotted lines*) and observed (*red* and *cyan*) at 120 and 200 cm, respectively. The periods designed Short (Sh) and Long (Lo) Rains are indicated in the space between the subplots. The simulation time period is 1 April 2012–31 December 2013. (Color figure online)

The variably saturated hydrologic flow simulation began 1 March 2012, and was calibrated to fit 7.5 weeks of observed soil moisture data from late 2013 (Fig. 4). Soil moisture at 120 and 200 cm increased by several percent following a series of rainy days in September 2013, early during the short rains (Fig. 4a). Deep soil moisture remained elevated for several weeks despite a decrease in precipitation. The fluid flow model simulated this sharp rise and slow decline in soil moisture content at depth (Fig. 4b). Physical flow parameters for soil layers determined from the calibrated model indicated that the upper model soil layer (0–100 cm) had the largest pore size distribution parameter, *λ*, and the lowest air entry pressure head, *h_b_* (Table 2). The layer with the highest clay content (100–150 cm) had the lowest pore size distribution and the largest air entry pressure head (Table 2). The calibrated model produced daily soil moisture content and fluid velocity from 1 March 2012 to 31 December 2013.

**Table 2:** VS2D model parameters: saturated hydraulic conductivity, *K_sat_*, residual moisture content, *θ_r_*, porosity, n, and the BrooksCorey parameters *h_b_* and *λ*

Layer depth (cm bgs)	*K_sat_*, (m day ^–1^)	*θ_r_*	*n*	*h_b_*	*λ*
0–100	0.1	0.065	0.41	−1.1	0.17
100–150	1	0.065	0.41	−0.5	0.12
150–500	1	0.038	0.38	−0.6	0.13

**Table 3:** Seasonal average soil water NO_3_^–^-N concentration (mg L^–1^) at two depths for the growing seasons (April 1–August 31)

Fertilizer application rate (kg-N ha ^–1)^	2012 growing season		2013 growing season		ΔNO_3_^–^-N (mg L^–1^)	
	120 cm	200 cm	120 cm	200 cm	120 cm	200 cm
0	7.9	11	16	9.3	7.6	−1.4
50	15	10	20	20	3.9	9.6
75	11	5.8	17	10	5.6	4.5
200	17	16	28	12	11	−3.5

Averages are calculated using daily concentrations based on Eq. 3. NO_3_^–^N is the average concentration in 2013 minus the average concentration in 2012

#### Leachate transport and timing

**Fig. 5:**
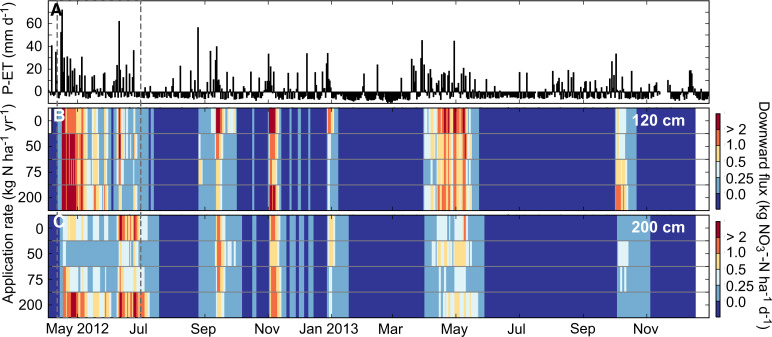
**a** Daily P-ET, **b** modeled daily downward NO_3_^–^-N flux for four fertilizer application rates at 120 cm, and **c** 200 cm. The magnitude of downward flux is shown from *dark blue (low)* to red (*high*). The *dashed gray box* corresponds to time period of data shown in Fig. 6. (Color figure online)

**Fig. 6:**
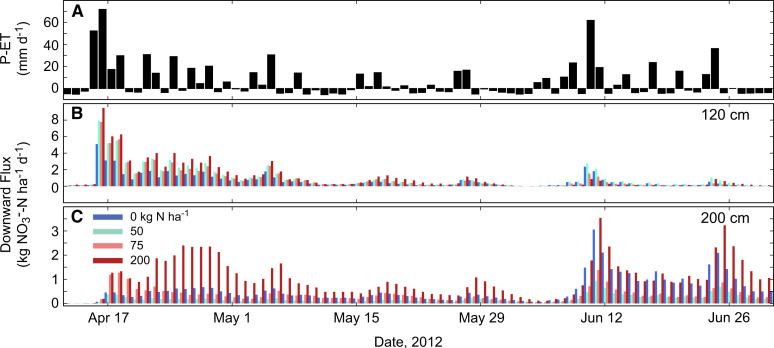
**a** Daily P-ET, **b** modeled daily downward NO^3^^–^-N flux for four fertilizer application rates from 12 April to 1 July, 2012, at 120 cm bgs, and **c** 200 cm bgs. Note the y-axis scale differs for plots B and C. (Color figure online)

Downward NO_3_^–^-N transport occurred during the rainy periods, while small upward soil solution transport (negative flux: 0 to –0.13 kg N ha^–1^ day^–1^ at 120 cm) occurred during the dry seasons (Fig. 5). At 120 and 200 cm, there was a lag of 1 and 2 days, respectively, before downward NO_3_^–^-N flux began at the start of the long rains in 2012 (Fig. 6), and a lag of 4 and 6 days, respectively, at the start of the long rains in 2013. The timing of transport lags may be used to inform future lysimeter monitoring schedules to better capture fluid front movement below the surface. During the long rains, the soil at 120 and 200 cm maintained saturation and downward soil solution flux, despite intermittent dry conditions at the surface.

The rewetting of dry soil at the beginning of the rainy season tends to cause a flush of NO_3_^–^-N in the topsoil known as the “Birch Effect” (Birch 1964), which reaches deeper soil layers as a delayed pulse of NO_3_^–^-N. This pulse of NO_3_^–^-N has been observed in clayey soils in SSA (Chikowo et al. 2004) and many other tropical regions (Hardy 1946). We observed this pattern but not consistently over the study period nor at both depths. At the start of the 2012 long rains, NO_3_-N leaching flux had a single day peak of 9.5 kg N ha^–1^ day^–1^ at 120 cm (Fig. 5). At 200 cm, leaching of up to 2.4 kg N ha^–1^ day^–1^ occurred within the first 2 weeks, however the seasonal peak (3.6 kg N ha^–1^ day^–1^) did not occur until 2 months later (Fig. 6). This temporal offset in peak flux rates was influenced by the time required to raise soil solution concentrations and to induce downward fluid flow in the subsurface. The rapid response at 120 cm and delayed, diffuse response at 200 cm may signify solute retardation processes in the deeper soil. During the 2013 long rains, which were notably less than 2012 (Table 4), the Birch Effect was not observed. Though the largest rain event (61 mm day^–1^) occurred early in the season, NO_3_^–^-N transport did not increase significantly until mid-season during a continuously wet period. Nitrate leaching flux peaked at 3.2 and 1.1 kg N ha^–1^ day^–1^ at 120 and 200 cm, respectively, in the middle of the 2013 growing season.

**Table 4:** Mean cumulative downward NO_3_^–^-N transport (kg NO_3_^–^-N ha^–1^) at two depths for the growing seasons (April 1–August 31) and full year (April 1–March 31)

Fertilizer application rate (kg-N ha^–1^)	2012 growing season		2012 full year		2013 growing season		Cumulative	
	120 cm	200 cm	120 cm	200 cm	120 cm	200 cm	120 cm	200 cm
0	62	46	90	65	33	12	123	77
50	73	41	97	66	25	13	122	79
75	60	26	68	40	31	13	98	53
200	93	49	138	81	22	14	160	95
Precip (mm)	1162		2303		760		3063	

Total precipitation is shown for each period

#### Seasonal NO_3_^–^-N leachate fluxes

Cumulative N flux over both growing seasons (1 April 2012–31 August 2013) ranged from 98 to 160 kg N ha^–1^ at 120 cm and 53–95 kg N ha^–1^ at 200 cm (Table 4). These rates are similar to those measured in Zimbabwe (Kamukondiwa and Bergstrom 1994; Mapanda et al. 2012), Kenya (Kimetu et al. 2006), and one study in Togo (Sogbedji et al. 2006). The range and maximum were both higher for another study at the same site in Togo using a different measurement method (Poss and Saragoni 1992). Average N leachate transport at 120 cm was 1.8 times higher than at 200 cm during the 2012 growing season, and 2.1 times higher in 2013. The decrease in N transport with depth was a function of N attenuation or removal in the soil column, and variations in fluid flow with depth. Conversely, between the growing seasons (1 September 2012–31 March 2013), NO_3_^–^-N leaching flux was higher at the deeper depth for fields receiving 50 and 75 kg N ha^–1^ year^–1^, though in all cases less than the flux estimated for the prior 2012 growing season.

The difference in leaching losses during a wet year (2012) and a drought year (2013) (Table 4) showed that reduced precipitation played a large role in reducing NO_3_^–^-N transport in soil solution. Though soil pore water NO_3_^–^-N concentrations were generally higher (Table 3), dry conditions resulted in less deep infiltration to transport nutrients beneath the root zone (e.g., Schmidt et al. 2004). Leachate fluxes during the 2013 growing season were 60% lower than during 2012 at 120 cm, and 65% lower at 200 cm (Table 4). Precipitation during the 2013 growing season was 35% lower than 2012, with a notably drier second half of the season (June to August). A review of field studies in Africa found that maize N uptake efficiency may be proportional to precipitation (Rufino et al. 2006), suggesting that lower crop uptake coupled with decreased fluid transport during droughts may lead to higher pore water concentrations and greater leaching in subsequent seasons. Additional years of observations would be required to determine how the seasonal total and pattern of precipitation controls the Birch Effect and cumulative nutrient flux across soils in this region.

Our NO_3_^–^-N leaching flux estimates were higher than for historically unfertilized sites (Andraski et al. 2000) and temperate clay-rich sites (Simmelsgaard 1998). Our estimates of NO_3_^–^-N leaching flux per unit area assumed constant concentration in space throughout the farm field. However, N fertilizer was applied only at the base of the plant and our lysimeters were located within plant rows; our NO_3_^–^-N leaching fluxes should be considered reasonable upper bounds. The average percent N loss by leaching was 50% in 2012, which was higher than the leachate loss in the similarly clayey soils of Zimbabwe (Mapanda et al. 2012). However, the percent N loss was much lower in the drier second study year (16%) and comparable to losses in other African clays (Kimetu et al. 2006; Mapanda et al. 2012).

#### Leachate rates from nitrogen fertilizer treatments

We found no significant correlation between N application and NO_3_^–^-N leaching over the study period, although plots receiving the highest N application (200 kg N ha^–1^ year^–1^) had the highest NO_3_-N leaching at 200 cm for all periods, and the highest at 120 cm for the 2012 growing season and between seasons. The mean coefficient of determination between N application and NO_3_^–^-N leaching from all the plots (R^2^ value) was 0.14 at 120 cm, though one of the trends is negative, and 0.038 at 200 cm. There was a lower correlation between N application and NO_3_^–^-N leaching for the three lower N application rates (mean R^2^ value was 0.04 and 0.007 for 120 and 200 cm depths, respectively). This contrasted with nitrous oxide emissions from the same plots, which increased proportionally to N application rate (Hickman et al. 2015). A study on clay-loam (New York State, USA) found similar N leaching rates for all application rates up to 100 kg ha^–1^ but then relatively higher for[100 kg ha^–1^ (Sogbedji et al. 2000), which was similar to, though not entirely consistent with our findings.

The observation of similar leachate loss from fields receiving 0, 50, and 75 kg N ha^–1^ fertilizer application was surprising and could have multiple explanations. First, these soils may have a high natural background N storage (Almaraz et al. in prep) and NO_3_^–^ flux rate through the surface 200 cm. Jégo et al. (2012) found the NO_3_^–^ leaching rate at 200 cm did not correlate with N application, but rather to initial soil N concentration. This is supported by the finding of relatively high amounts of exchangeable NO_3_^–^-N in these soils to 400 cm with similar concentrations across all plots (279 kg N ha^–1^; Tully et al. 2016). While the source of the N moving below 200 cm remains unknown, the substantial and consistent decrease in NO_3_^–^-N concentration and flux between 120 and 200 cm suggests strong soil NO_3_^–^-N retention and some of this N potentially could be a source for leached N under future conditions.

Second, because our experimental field had not received N fertilizer for many years, it is possible that a relationship between N application and N leaching would develop after additional years of N fertilizer application as soils reached a new equilibrium. As the ion exchange sites are filled, the ability of the soil to retain excess N may decrease. For example, on a siltloam field (Wisconsin, USA) with no history of N fertilizer application, Andraski et al. (2000) found total NO_3_^–^ leaching losses increased from 21 to 32 kg N ha^–1^ between the first and second experiment years, respectively. After multiple years of N fertilizer application, NO_3_^–^-N concentration in the soil solution may rise proportionally to excess N application rate (e.g., Perego et al. 2012).

Because interactions between soil properties, N application timing, and precipitation patterns controlling retention and leaching likely also occur below 200 cm, behavior through the deep (>4 m) soil -column will ultimately control NO_3_^–^ losses at the watershed scale. The soil in Kenya is deep and clayey to depth so the estimates of leaching at 200 cm may not reflect near-term leaching into water sources. Measurements at additional depths throughout more seasons would be needed to develop a predictive model of N transport and storage for the region.

### Site model calibration and limitations

We modeled only fluid flow rather than including solute transport using an advection–dispersion equation (ADE) model. Numerous flow and transport models exist and are commonly applied for lysimeter studies, however we elected not to model solute transport because of uncertainties in the characteristics of our system, including N cycling processes, physical flow dynamics, and concerns with application of the ADE without appropriate parameter data (Konikow 2011). Quantifying the fluid transport rate, as done in this study, is a prerequisite for any solute transport model, which could be pursued with additional field data collection and modeling in the future.

VS2D simulates variably saturated fluid flow, and solute or heat transport, but does not simulate plant growth. Though generally consistent soil moisture and texture conditions between plots supported using a single model for fluid transport within all fertilizer treatments, this does not account for differences in crop water uptake between the high and low yielding plots. In addition, the VS2D model does not explicitly simulate macropore and matrix flow, but rather the average total flow. The model of our study area could be improved by accounting for dual porosity flow using for example the dual porosity flow model MACRO + SOILN (e.g., Larsson and Jarvis 1999), or RZWQM2 (e.g., Nolan et al. 2010), but would require additional field measurements to calibrate the additional transport process. Another model design limitation was caused by the disparity between model time step and the pattern of rainfall in the study area. Rainfall at the site tends to occur in short, intense events, with more than 70% of total daily rain occurring with in 1 h of the day. The model uses daily time-steps for computational reasons, which can lead to a representation of less intense rain falling evenly throughout each day. On low permeability surface soils, this may lead to under-representation of runoff on days with large intense rain events (Germer et al. 2010), or conversely an under-representation of infiltration on days when rainfall is of similar magnitude as ET. These are common model limitations; addressing them in future work will depend on balancing field resources with model capability.

## Conclusion

We combined pore water nutrient measurements and a numerical fluid flow model to calculate high temporal resolution estimates of NO_3_^–^-N leaching flux at 120 and 200 cm depths from soils that received from 0 to 200 kg N ha^–1^ year^–1^ fertilizer. The NO_3_^–^-N flux at both depths followed a similar pattern of increase following N fertilizer application and again following harvest. Nutrient fluxes were approximately twice as high at 120 cm compared to 200 cm during the growing seasons. There was no significant correlation between N application and leaching flux, though the fluxes were highest at 200 cm for both seasons. The absence of a proportional increase in NO_3_^–^-N fluxes on plots receiving applied N suggested significant N storage capacity in these tropical soils. Relatively high and consistent NO_3_^–^-N fluxes to 200 cm suggested that processes that control NO_3_^–^-N retention and leaching in these deep (>4 m) soils will regulate NO_3_^–^-N losses at the watershed scale. During the drought season with 30% less rain than the previous year, NO_3_^–^-N pore water concentrations were higher, while leaching was ~60% lower; this indicated that annual precipitation exerted a large control on annual NO_3_^–^-N flux. Subsequent years of NO_3_^–^-N concentration monitoring and modeling on deep, tropical soils would be needed to quantify and predict long-term consequences of higher annual N fertilizer applications. Further study of the fate of applied N in SSA over time are needed to develop fertilizer application recommendations optimized to mitigate potential NO_3_^–^-N losses from farm fields while meeting food security demands.
